# What is done when endoscopic examination reveals borderline bowel ischemia in patients with sigmoid volvulus?

**DOI:** 10.12669/pjms.333.12265

**Published:** 2017

**Authors:** Sabri Selcuk Atamanalp, Refik Selim Atamanalp

**Affiliations:** 1 Prof. Sabri Selcuk Atamanalp, MD. Department of General Surgery Faculty of Medicine, Ataturk University, Erzurum, Turkey; 2 Refik Selim Atamanalp, English Medicine Program, Faculty of Medicine, Ataturk University, Erzurum, Turkey

**Keywords:** Sigmoid Colon, Volvulus, Endoscopy, Ischemia, Treatment

## Abstract

Sigmoid volvulus (SV) is a rare colonic obstruction in which the sigmoid colonwraps around itself. The principal strategy for the treatment of uncomplicated SV is emergency endoscopic detorsion, while emergency surgery is needed in complicated SV with bowel gangrene, bowel perforation, peritonitis, or unsuccessful endoscopic treatment. In the endoscopic examination of SV, endoscopic detorsion is performed if the bowels are viable, while emergency surgery is needed if the bowels are gangrenous. However, the treatment approach is not clear when endoscopic examination reveals borderline bowel ischemia, and in this short report, the possible treatment strategies are discussed.

## INTRODUCTION

SV is the wrapping of the sigmoid colon around its base, which causes acute mechanical large bowel obstruction.^1^ SV is an uncommon disease, but its incidence is relatively high in African, Asian, Middle Eastern, South American, and Eastern and Northern European countries,^1^ as well as in Turkey, particularly in Eastern Anatolia,^2^-^4^ where our hospital is located.

After resuscitation, clinical examination and radiological screening, the preferred approach to the emergency treatment of uncomplicated SV patients is endoscopic detorsion, while emergency surgery is needed for complicated patients in whom bowel gangrene, bowel perforation, peritonitis, or unsuccessful endoscopic treatment has occurred.^1^,^3^,^5^-^8^ Endoscopy both assists with diagnosis by showing a spiral sphincter-like twist of the obstructive lumen 20-30 cm from the anal verge and allows direct visualization of the bowel mucosa viability.^3^,^4^,^6^,^8^ Bowel gangrene is a catastrophic complication of SV that develops in 6.1-93.4% of SV cases and doubles the mortality rate.^4^,^9^ Endoscopic signs of gangrene include devitalized mucosa following detorsion and gangrenous bloodstained effluent from the endoscope.^4^,^5^,^9^ The therapeutic strategy in patients with SV in whom endoscopic examination demonstrates viable bowel or bowel gangrene is well established, and if the viability of the bowel is sufficient, the treatment is endoscopic detorsion if possible, while resection of the gangrenous segments by surgery is needed in cases in with the bowel is gangrenous.^1^,^4^,^5^-^8^ Otherwise, there is no available data in the literature to indicate the treatment approach when borderline bowel ischemia is observed in the endoscopic examination.^1^,^3^-^8^,^10^-^12^

In this preliminary report, we observed 4 of 7 patients with borderline bowel ischemia treated in the last 10 years of our 50.5-year clinical experience, during which time a total of 997 patients and 721 endoscopically treated patients were treated. These four patients were treated by performing a repeat endoscopic examination after the endoscopic detorsion, and we evaluated this therapeutic strategy.

### What can be done in cases of borderline Bowel Ischemia

As previously discussed, unfortunately, there are no available data in the literature on this subject.^11^,^12^ In our opinion and experience, although most such cases are treated with emergency surgery, as is appropriate for patients with gangrenous bowel, there are three options. The first choice is surgical treatment without endoscopic detorsion, which may be applied in most of cases without unstable vital functions and/or major comorbidity risk for a major emergency operation under general anesthesia. In this approach, the patients are subjected to an emergency laparotomy, and based on the laparotomy findings and patient conditions, one of the resectional or nonresectional surgical procedures is selected. If bowel gangrene is present, after the resection of gangrenous segments, primary anastomosis is preferred, while stoma is a lifesaving procedure in patients with unstable vital functions, peritonitis, and major comorbidity. If the bowels are viable, resection with primary anastomosis is also used in selected patients, while detorsion alone, or with some recurrence preventing procedures such as mesopexy or mesoplasty, may be applied.^1^,^3^,^5^-^8^,^10^ The second modality is, following endoscopic detorsion, the observation of the patients through careful monitoring, including vital functions, clinical examination, and ischemia parameters. In this approach, the treatment is directed according to the results of the criteria mentioned above. Additionally, if possible, advanced diagnostic techniques such as ultrasonography,^13^ angiography^14^ or scintigraphy^15^ may be used in the determination of the bowel vascularization and viability. The last method is performing a second look endoscopy after an observation period of a few hours. In this approach, a repeated endoscopic examination is performed after 6-8 hours, in which the bowel viability may be evaluated as healthier due to the end of the adverse effects of the torsion and obstruction on vascularization, and the treatment is directed according to the endoscopy findings. If needed, a third look endoscopy may also be performed in selected patients in whom the bowel viability is not evaluated accurately in the second examination.

## CLINICAL EXPERIENCE

Our clinic has seen a total of 997 patients and 721 endoscopically treated patients with SV over a 50.5-year-period between July 1966 and January 2017. Although, like most surgeons, we preferred emergency surgery in the evaluation and treatment of borderline ischemic cases in the past, we have evaluated seven patients with borderline bowel ischemia in the last 10 years. Three of them (42.9%) were treated by emergency surgery, and sigmoid resection with primary anastomosis was applied. Four other patients (57.1%) were observed, and a second look endoscopic examination was performed after 6-8 hours. In one of them (25.0%), a delayed emergency surgical sigmoid resection with primary anastomosis was performed due to the development of bowel gangrene, while the other three patients (75.0%) were advised to receive elective surgery. The two who accepted the surgery were treated by elective sigmoid resection with primary anastomosis after a few days. None of the seven patients were lost, and wound infection developed in one patient (33.3%) in the emergency surgery group. This work is the first preliminary report on this subject.^11^,^12^

## DISCUSSION

To determine an algorithm for the assessment and treatment of SV patients with borderline bowel ischemia in endoscopic examination, we reviewed the literature in major research databases, including Web of Science^11^ and PubMed.^12^ Interestingly, we could not find any data or discussion material on this subject. Therefore, we evaluated our clinical records of SV patients, as well as our experiments, in the largest single-center SV series of the world.^2^-^4^,^11^,^12^ In our opinion, there are three options: emergency surgical treatment rounding out endoscopy, endoscopic detorsion followed by observation with the use of advanced diagnostic techniques, and repeated endoscopic examination following endoscopic detorsion. The first option, emergency surgical treatment, has the heightened morbidity and mortality risk of a major operation, particularly in unstable, contaminated and comorbid patients.^1^,^3^,^5^-^8^,^10^ Although the most important disadvantage of this approach is that instead of a successful endoscopic detorsion followed by elective surgery,^16^,^17^ an emergency surgery with higher morbidity and mortality is performed,^1^,^3^,^5^-^8^,^10^ it is the treatment approach that we preferred in the past, like most surgeons. The second option, observation with clinical and laboratory evaluation and, if possible, using Doppler ultrasonography, catheter or CT angiography, or scintigraphy to evaluate a further consideration of the bowel vascularization and viability, does not seem practicable due to the inability of the routine clinical and laboratory ischemia parameters to accurately diagnose ischemia,^1^,^2^,^4^,^9^ in addition to the uncommon use of some of those advanced techniques and the corresponding inexperience with their usage in SV.^11^,^12^ Although it is possible to evaluate the lower intestinal vascularization as well as several other diseases, including bleeding, using those techniques, and experiments have been performed,^13^-^15^ there is no report of their usage in sigmoid volvulus in the literature,^11^,^12^ which is similar to our own inexperience. The probability of missing bowel gangrene and/or the development of toxic shock during an observation period of a few hours or days represents another disadvantage of this approach. For these reasons, the most appropriate option seems to be the last: performing a repeating endoscopy 6-8 hours after the endoscopic detorsion to obtain a safer evaluation of the bowel viability. If the first endoscopic examination is possible, a repeating one is also applicable. Our experience, although limited, supports this practice. In our opinion, this approach is a good alternative to emergency surgery in units in which experienced endoscopists are present, and it is the option that we have preferred in the recent past.

## CONCLUSION

We recommend endoscopic detorsion followed by repeated endoscopic examination in the evaluation and treatment of selected patients with SV whose endoscopic examination demonstrates borderline bowel ischemia. Patients should be hospitalized and carefully monitored by an experienced endoscopy team. However, further studies are needed to confirm our findings.

### Authors’ contribution

***SSA*** designed the study, collected and analysed the data, reviewed the literature and prepared the manuscript.

***RSA*** reviewed the literature and approved the final version of the manuscript.
